# Characterization of mesothelin gene expression in dogs and overexpression in canine mesotheliomas

**DOI:** 10.3389/fvets.2024.1436621

**Published:** 2024-09-09

**Authors:** Rina Nabeta, Ami Kanaya, Kazumi Shimada, Katsuhiro Matsuura, Aritada Yoshimura, Tomohiro Oyamada, Daigo Azakami, Tetsuya Furuya, Tsuyoshi Uchide

**Affiliations:** ^1^Laboratory of Veterinary Molecular Pathology and Therapeutics, Faculty of Agriculture, Tokyo University of Agriculture and Technology, Fuchu, Japan; ^2^Laboratory of Veterinary Surgery, Faculty of Agriculture, Tokyo University of Agriculture and Technology, Fuchu, Japan; ^3^Department of Small Animal Clinical Science, College of Veterinary Medicine, University of Florida, Gainesville, FL, United States; ^4^Animal Medical Center, Faculty of Agriculture, Tokyo University of Agriculture and Technology, Fuchu, Japan; ^5^Laboratory of Veterinary Clinical Oncoogy, Faculty of Agriculture, Tokyo University of Agriculture and Technology, Fuchu, Japan; ^6^Laboratory of Veterinary Infectious Diseases, Faculty of Agriculture, Tokyo University of Agriculture and Technology, Fuchu, Japan

**Keywords:** sequence analysis, biomarker, megakaryocyte potentiating factor, cancer, immunotherapy, effusion, tissue, diagnosis

## Abstract

**Introduction:**

Canine mesotheliomas are uncommon malignant tumors typically detected late. Minimally invasive diagnostic biomarkers would facilitate diagnosis at earlier stages, thereby improving clinical outcomes. We hypothesized that mesothelin could be used as a reliable diagnostic biomarker for canine mesotheliomas since it has been used as a cancer biomarker for human mesothelioma. We aimed to explore and characterize mesothelin gene expression in dogs and assess its use as a diagnostic biomarker for canine mesotheliomas.

**Materials and methods:**

We quantified expressed canine mesothelin transcripts via reverse transcription polymerase chain reaction (RT-PCR) and sequenced them using ribonucleic acid (RNA) extracted from a canine mesothelioma cell line. After confirming mesothelin expression, we assessed its levels in major organ tissues and compared them with those in the mesothelioma tissues using quantitative PCR (qPCR). Mesothelin overexpression in mesotheliomas was detected, and we further compared its levels using qPCR between mesotheliomas and non-mesotheliomas using tumor tissues and clinical sample effusions, confirming its significance as a diagnostic biomarker for canine mesothelioma.

**Results:**

Mesothelin complementary deoxyribonucleic acid (cDNA) was amplified via RT-PCR, yielding a single band of expected upon DNA electrophoresis. Sequence analyses confirmed it as a predicted canine mesothelin transcript from the genome sequence database. Comparative sequence analysis of the deduced amino acid sequence of the expressed canine mesothelin demonstrated molecular signature similarities with the human mesothelin. However, the pre-sequence of canine mesothelin lacks the mature megakaryocyte potentiating factor (MPF) portion, which is typically cleaved post-translationally with furin. Mesothelin expression was quantified via qPCR revealing low levels in the mesothelial and lung tissues, with negligible expression in the other major organs. Canine mesothelin exhibited significantly higher expression in the canine mesotheliomas than in the noncancerous tissues. Moreover, analysis of clinical samples using qPCR demonstrated markedly elevated mesothelin expression in canine mesotheliomas compared to non-mesothelioma cases.

**Discussion and conclusion:**

Canine mesothelin exhibits molecular and biological characteristics akin to human mesothelin. It could serve as a vital biomarker for diagnosing canine mesotheliomas, applicable to both tissue- and effusion-based samples.

## 1 Introduction

Mesotheliomas are rare malignant tumors, representing about 0.1–0.2% of canine tumors. They arise from the mesothelial cells, which form a cell monolayer called the mesothelium. The mesothelium lines the surfaces of body cavities, such as the pericardial, pleural, and peritoneal cavities. The classical clinical signs of mesothelioma include recurrent refractory effusion in the affected body cavities, dyspnea, and cardiac tamponade secondary to effusion (1–3). Such effusions usually contain various numbers of neoplastic mesothelial cells exfoliated from the original tumor; thus, mesotheliomas readily spread throughout the body cavities (4, 5). Mesotheliomas often invade the surrounding tissues along with the serous membrane in addition to the rapid spread of tumors in the body cavities and usually do not develop into a discrete large mass that can be detected via imaging diagnostic modalities (5, 6). Mesotheliomas are most often diagnosed at advanced stages owing to the aggressive nature of tumors and their unique pattern of tumor growth. Hence, curative interventions such as complete surgical excision are no longer amenable, and the efficacy of alternative treatments, including chemotherapy, is not guaranteed (3, 7). By increasing the chances of detecting malignancy minimally invasive diagnostic biomarkers would facilitate definitive diagnoses at earlier stages, thereby playing a critical role in combating this malignant tumor.

In human medicine, extensive research has been conducted to achieve an early diagnosis using reliable mesothelioma biomarkers. Among the several discovered biomarker candidates, mesothelin, a cell-surface membrane-bound glycophosphatidylinositol (GPI)-anchored protein, is the only molecule approved by the Food and Drug Administration for clinical use in mesothelioma (8–11). Mesothelin is a peculiar molecule, as its expression is limited to only a few organs in health, with low expression found in the mesothelial tissues, whereas overexpression has been detected in mesothelioma (8, 10–15). However, no useful biomarker is currently available in veterinary medicine, and substantial studies on mesothelin in dogs are absent.

We hypothesized that mesothelin could act as a reliable diagnostic marker for canine mesotheliomas based on the biological similarities between human and canine mesotheliomas. The study objectives included (1) confirming the gene expression of mesothelin in dogs (2), characterizing the expression pattern of mesothelin (3), evaluating mesothelin overexpression with mesotheliomas, and (4) evaluating and comparing the mesothelin expression between dogs with mesotheliomas and those with non-mesothelioma diseases using clinical samples. We aimed to detect and characterize mesothelin gene expression in dogs and assess its diagnostic usefulness in canine mesotheliomas.

## 2 Materials and methods

### 2.1 Sample collection

#### 2.1.1 Culture cells

In this study, primary cultures of the canine pericardial mesothelioma previously generated in our laboratory (16) and the cell line MC18003 were used. The cell line was derived from the pleural effusion collected from a canine patient diagnosed with pericardial mesothelioma via histopathology.

#### 2.1.2 Tissues from tumor-free dogs

Specimens of the organ tissues, including the pericardium, pleura, liver, spleen, kidney, heart, lung, and pancreas (*n* = 24 in total, three each), were obtained from four beagle dogs (two female and two male) aged 1–2 years of age under general anesthesia using intravenous propofol (5–6 mg/kg, Fresenius Kabi) and inhalational isoflurane (1.2–1.7% endo-tidal concentration, Pfizer) as described in the original study (17). Dogs were originally used as models of tachycardia-induced cardiomyopathy, and we used the remaining tissue samples. After physical examination, complete blood count, blood chemistry, radiography, and ultrasonography, the dogs were initially deemed healthy before the procedure to create a tachycardia state. The tissue specimens were trimmed using surgical instruments to approximately 0.5 cm^3^ in size. The collected tissues were immersed in 0.5–1.0 mL RNA later™ Stabilization Solution (RNA later) (Invitrogen) overnight at 4°C and stored at −80°C until use. The Tokyo University of Agriculture and Technology Animal Experiment Committee (approval No. 31–2) approved all experimental procedures, which were performed in accordance with the recommendations of the guidelines released by the committee.

#### 2.1.3 Clinical samples

Clinical samples of the tumor tissues and effusions were collected from the canine patients (40 dogs in total; 12 castrated male, 16 spayed female, 7 male, 5 female; 2–18 years of age; 2 Miniature Dachshund, 1 Miniature Bull Terrier, 2 Toy Poodle, 1 Shih Tzu, 3 Yorkshire Terrier, 4 Chihuahua, 1 Miniature Schnauzer, 1 Border Collie, 2 Jack Russell Terrier, 1 Papillon, 1 American Cocker Spaniel, 3 Welsh Corgi, 2 Pomeranian, 1 Akita, 1 Flat-coated Retriever, 1 Maltese, 1 Shiba Inu) between 2018 and 2023. An attending veterinarian obtained written or verbal informed consent from the owners of all dogs included in this study. For treatment and diagnostic purposes, the tumor tissues were surgically removed. Tissue specimens (0.5 cm^3^ in size) were collected from residual tumor tissue after trimming for histopathology submission. The specimens were treated in similarly as described above and subsequently immersed in RNA later. The diagnoses of all tumors were confirmed via histopathology. The effusion samples were removed in a sterile manner using an ultrasound-guided technique. Effusion sediments were immediately isolated after collection via centrifugation at 600 × *g* for 3 min and washed three times in phosphate-buffered saline. A sample having prominent blood contamination was incubated with red blood cell (RBC) lysis buffer (Sigma Life Science) for 15 min at 37°C to lyse the red blood cells. Following the final centrifugation, 0.5–1.0 mL of RNA later was added to the sediments. The samples were kept at 4°C overnight, and stored at −80°C until further analysis. Table 1 shows the signals and final diagnoses of the clinical cases.

**Table 1:** Signalment and the diagnosis of the clinical cases involved in this study.

ID	Sample type	Breed	Sex	Age (years old)	Dianogis
409	Tissue	Miniature Dachshund	Castrated male	10	Mesothelioma
499	Tissue	Miniature Bull Terrier	Spayed female	8	Mesothelioma
660	Tissue	Toy Poodle	Male	12	Mesothelioma
718	Tissue	Shih Tzu	Male	11	Mesothelioma
1	Tissue	Yorkshire Terrier	Male	13	Seminoma
4	Tissue	Yorkshire Terrier	Male	13	Leydig cell tumor
78	Tissue	Mix	Castrated male	11	Myxosarcoma
83	Tissue	Mix	Female	13	Mammary gland adenoma
87	Tissue	Chihuahua	Spayed female	11	Hepatocellular carcinoma
93	Tissue	Toy Poodle	Female	13	Mammary gland adenoma
96	Tissue	Mix	Male	18	Malignan melanoma
103	Tissue	Chihuahua	Spayed female	12	Liposarcoma
105	Tissue	Yorkshire Terrier	Spayed female	13	Hepatocellular carcinoma
113	Tissue	Miniature Schnauzer	Spayed female	11	Histiocytic sarcoma
118	Tissue	Mix	Castrated male	11	Apocrine gland adenocarcinoma of anal sac
122	Tissue	Mix	Castrated male	12	Trichoepithelioma
163	Tissue	Border Collie	Female	12	Leiomyoma
210	Tissue	Jack Russell Terrier	Spayed female	11	Hepatocellular carcinoma
214	Tissue	Mix	Castrated male	12	Canine perivascular wall tumor
218	Tissue	Mix	Castrated male	12	Hepatocellular carcinoma
223	Tissue	Papillon	Castrated male	9	Lipoma
230	Tissue	American Cocker Spaniel	Spayed female	14	Sebaceous carcinoma
232	Tissue	Welsh Corgi	Spayed female	11	Canine perivascular wall tumor
238	Tissue	Welsh Corgi	Castrated male	9	Trichilemmoma
245	Tissue	Mix	Male	8	Hepatocellular carcinoma
251	Tissue	Mix	Spayed female	10	Leiomyosarcoma
259	Tissue	Mix	Spayed female	15	Hepatocellular carcinoma
262	Tissue	Pomeranian	Female	14	Apocrine gland adenocarcinoma of anal sac
257	Pleural effusion	Yorkshire Terrier	Castrated male	6	Mesothelioma
454	Pleural effusion	Miniature Bull Terrier	Spayed female	8	Mesothelioma
507	Pericardial effusion	Miniature Bull Terrier	Spayed female	8	Mesothelioma
571	Pericardial effusion	Jack Russell Terrier	Female	Unknown	Mesothelioma
655	Pleural effusion	Toy Poodle	Male	12	Mesothelioma
656	Pericardial effusion	Toy Poodle	Male	12	Mesothelioma
19,002	Pleural effusion	Miniature Dachshund	Castrated male	10	Mesothelioma
23,180	Pleural effusion	Akita	Castrated male	12	Mesothelioma
23,202	Pleural effusion	Chihuahua	Spayed female	13	Mesothelioma
150	Pleural effusion	Welsh Corgi	Spayed female	12	Mammary gland carcinoma
311	Pleural effusion	Pomeranian	Spayed female	17	Cardiogenic pulmonary edema
383	Pericardial effusion	Flat-coated Retriever	Castrated male	9	Pericarditis
447	Pleural effusion	Mix	Male	14	Chemodectoma
585	Pericardial effusion	Miniature Dachshund	Male	15	Lymphoma
621	Peritoneal effusion	Mix	Spayed female	13	Protein losing enteropathy
631	Pleural effusion	Maltese	Spayed female	15	Hypertrophic cardiomyopathy
670	Pleural effusion	Shiba Inu	Castrated male	2	Chylous
680	Pleural effusion	Chihuahua	Spayed female	13	Inflammatory bowl disease, Pancreatitis
689	Peritoneal effusion	Mix	Castrated male	13	Pancreatitis

Animal ethics approval was obtained to collect the clinical tumor tissue and effusion samples, and the entire study was conducted following the recommendations of the Guidelines followed by the Clinical Research Ethics Committee of the Tokyo University of Agriculture and Technology (approval No. 0016017, 0020005, 00500011).

### 2.2 Database search

Canine and human mesothelin genes (Gene ID LOC611363) were searched in the National Center for Biotechnology Information (NCBI) gene database. The canine reference used was the genomic sequence of the ROS_Cfam_1.0 Assembly (NC_051810.1) to obtain the genetic information and assess structural characteristics. Human mesothelin protein features were analyzed using the UniProt database (Q13421).

### 2.3 Total RNA extraction

Total RNA was extracted from the cultured cells, effusion sediments, and tissues using NucleoSpin RNA (Macherey-Nagel), according to the manufacturer’s instructions. Briefly, the tissue samples were minced and homogenized using a scalp blade, surgical scissors, and an 18-gauge needle. Subsequently, the samples were lysed in a buffer containing 1 μL 1 M dithiothreitol (DTT) as a reducing agent. The total RNA was bound to the membrane of the column after filtering the lysate. The genomic DNA was digested using DNase at room temperature for 15 min. Total RNA was eluted in 60–120 μL nuclease-free water. The elution was aliquoted in three tubes and stored at −80°C until further analysis.

### 2.4 Reverse transcription polymerase chain reaction (RT-PCR)

Total RNA extracted from the cultured cells (MC18003) was transcribed into cDNA using ReverTra Ace qPCR RT Master Mix (Toyobo). Subsequently, RT-PCR was conducted using GoTaq® Green Master Mix (Promega) to amplify the cDNA of the canine mesothelin with the following conditions: 95°C for 4 min for the initial denaturation, 35 cycles of 95°C for 30 s, 61°C for 30 s, and 72°C for 60 s for amplification, and 72°C for 7 min for the final extension. Custom designed primer pair was employed for the reaction and the primer sequences were 5′ GAG ACA AGA GAA GGC CAG TCC 3′ for a forward primer and 5′ CCC TGG CCA CCC AAA TAA CAC 3′ for a reverse primer. The primer pair specificity was confirmed using the NCBI Primer- Basic Local Alignment Search Tool (BLAST). The predicted targets included canine mesothelin variants 1–4 and 8–9, according to the NCBI canine genomic database. To verify their sizes, amplicons were subjected to gel electrophoresis at 100 V for 30 min. The predicted amplicon sizes were 537 bp for variants 1 and 8–9, 534 bp for variant 2, 477 bp for variant 3, and 474 bp for variant 4. RT-PCR products were subjected to sequence analysis in a commercial laboratory. The sequences obtained were referenced to the NCBI database.

### 2.5 Quantitative PCR (qPCR)

To compare the relative expression of the mesothelin mRNA among the major organ tissues in dogs, qPCR was performed using an Applied Biosystems StepOne™ Real-Time PCR System (Thermo Fisher Scientific). Additionally, to investigate the potential usefulness of mesothelin as a diagnostic biomarker for canine mesotheliomas, the relative mesothelin expression levels in the tissues and effusions were compared between the mesotheliomas and non-mesotheliomas. THUNDERBIRD® Probe One-step qRT-PCR Kit (Toyobo) was used for the qPCR reaction. The internal control selected based on previous studies (18, 19) was hypoxanthine phosphoribosyltransferase 1 (HPRT1), a housekeeping gene. Predesigned TaqMan probe and primer sets (TaqMan Gene Expression Assay, Thermo Fisher Scientific) for mesothelin (Cf02661438_m1) and HPRT1 (Cf02690456_g1) were used. All samples were run in triplicates for technical replication. Data analysis was conducted using StepOne v2.3 (Thermo Fisher Scientific), and the relative expression of mesothelin to HPRT1 was compared using the delta Ct method (20).

### 2.6 Statistical analysis

The mean and standard error of the Ct values were calculated using the software, and the data are shown relative to HPRT1. For experiments using clinical samples, the differences between groups were examined using the Mann–Whitney test with Bonferroni correction when applicable for statistical analysis. Statistical significance was set at *p* < 0.05 (20).

## 3 Results

### 3.1 Clinical samples

A total of 28 tumor tissue specimens, including four mesotheliomas, were collected. Nineteen effusion samples were obtained, of which 9 were from dogs diagnosed with mesothelioma. Table 1 lists the sample types, signalments, and diagnoses.

### 3.2 RT-PCR and sequence analysis

**Figure 1:**
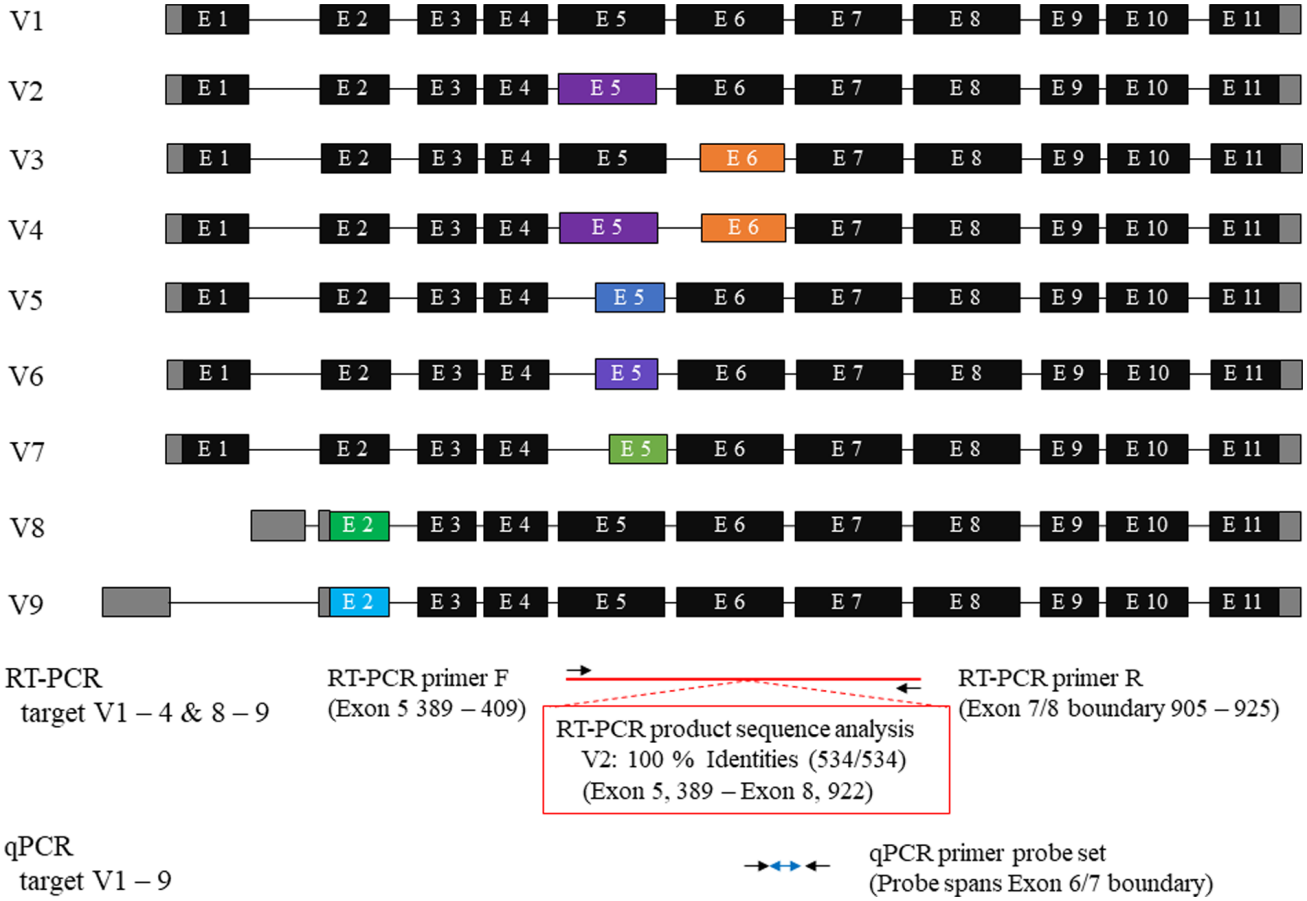
Canine mesothelin variants and primer designs. A database search showed nine canine mesothelin splicing variants. Canine mesothelin splicing variants are likely a product of alternative selection of the 3′ or 5′ splice sites. The target region for reverse transcriptase-polymerase chain reaction (RT-PCR) was exons 5–8, with the reverse primer designed to span the exon boundary. The amplicon sequence shared 100% identity with the canine mesothelin variant 2 (XM_038669678). Predesigned TaqMan probes and primer sets for mesothelin, designed to cover all variants, were used for quantitative PCR (qPCR); therefore, allowing evaluation of the changes in the overall mesothelin gene expression, irrespective of the variant that was considered biologically crucial.

**Figure 2:**
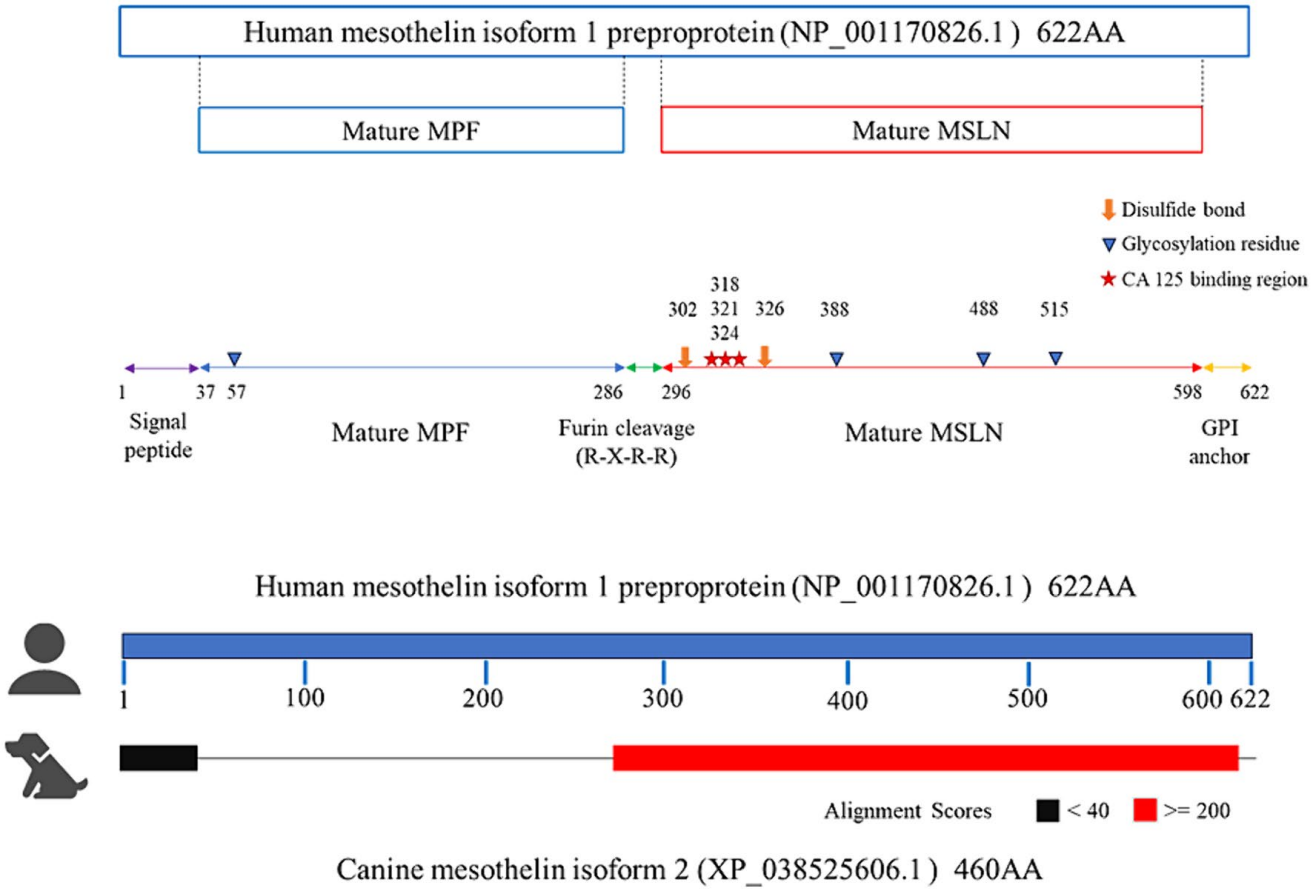
Comparing the amino acid sequences of the canine and human mesothelin. In the region where mature mesothelin is coded (red), canine mesothelin isoform 2 (XP_038525606), encoded by its variant 2, demonstrated substantial (70.34%) sequence identity with the human mesothelin isoform 1 (NP_001170826), the predominant isoform of mesothelin in humans. Comparison of canine and human mesothelin amino acids sequence revealed canine mesothelin could have a potential signal peptide (residues 37–69, corresponding to residues 1–37 of human mesothelin depicted in purple line), furin protease cleavage site (a predicted cleavage site at Arginine^109^, corresponding to Arginine^295^ of human mesothelin), disulfide bond (Cysteine^139^ and Cysteine^163^, corresponding to Cysteine^302^ and Cysteine^326^ of human mesothelin depicted in orange thick arrow), glycosylation residues (N-linked glycan at Asparagine^225^ and Asparagine^352^, corresponding to Asparagine^388^ and Asparagine^515^ of human mesothelin depicted in blue arrowhead), and GPI-anchorage attachment site (a predicted cleavage site at Serine^435^, corresponding to Serine^598^ of human mesothelin). Moreover, functionally important amino acids in human mesothelin (starts; tyrosine^318^, tryptophan^321^, glutamic acid^324^) were all conserved in canine mesothelin (tyrosine^155^, tryptophan^158^, glutamic acid^161^).

RT-PCR was performed using total RNA extracted from primary mesothelioma cultures to confirm mesothelin gene expression in dogs. The primary culture was obtained from a dog with spontaneous pericardial mesothelioma. Agarose gel electrophoresis revealed a band with a predicted size of approximately 530–540 bp. Sanger sequencing was conducted in a commercial laboratory and data analysis was successful for a length of 534 bp. Blast searching confirmed that the amplicon was consistent with canine mesothelin, which has 9 splicing variants. The sequence shared 100% identity with the canine mesothelin variant 2 (XM_038669678) and 99.44% identity with the variants 1, 8, and 9 (Figure 1). The supplementary material provides the BLAST description table (Supplementary Table S1). Canine mesothelin isoform 2 (XP_038525606), encoded by its variant 2, demonstrated substantial (70.34%) sequence identity with human mesothelin isoform 1 (NP_001170826), the predominant isoform of mesothelin in humans, in the region where mature mesothelin is encoded (21) (Figure 2) (Supplementary Figure S1). A comparison of the canine and human mesothelin amino acid sequences showed that the canine mesothelin has a putative signal peptide, a protease cleavage site, a disulfide bond, sequence glycosylation residues, and a GPI-anchorage attachment site. The GPI-anchorage sequence does not have a canonical sequence; instead, it exhibits common characteristic features (22, 23). In addition to the structural similarities, functionally important amino acids in the human mesothelin (tyrosine^318^, tryptophan^321^, and glutamic acid^324^) were conserved in the canine mesothelin. Notably, the pre-sequence of canine mesothelin lacks the N-terminal region corresponding to the human megakaryocyte potentiating factor (MPF).

### 3.3 Mesothelin expression in dogs without tumors

**Figure 3:**
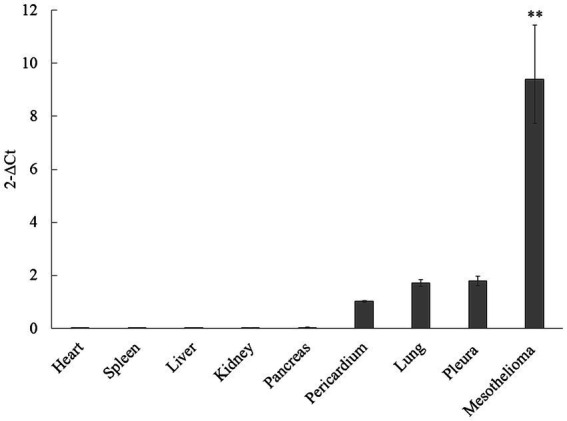
Mesothelin expression distribution in dogs and overexpression in canine mesotheliomas. The mesothelin expression distribution in dogs was analyzed using quantitative polymerase chain reaction (qPCR). Mesothelin expression was low in the mesothelial and lung tissues, whereas only negligible expression was observed in the other major organ tissues. Mesothelin was overexpressed (*p* < 0.001) in the mesothelioma cells.

Mesothelin gene expression levels in major organ tissues (*n* = 24) and mesothelioma tissues (*n* = 4) were analyzed via qPCR relative to the housekeeping gene HPRT-1 to characterize mesothelin’s tissue distribution and assess its expression in canine mesotheliomas (Figure 3). Substantial mesothelin expression was detected in the pleura (mean, 1.78; 95% confidence interval [CI], 1.60–1.98), lung (mean, 1.70; 95% CI, 1.59–1.84), and pericardial (mean, 1.02; 95% CI, 0.98–1.06) tissues, whereas other major organs demonstrated negligible mesothelin expression (mean, < 0.1). Additionally, the mesothelioma tissues exhibited significantly higher mesothelin levels (mean, 9.40; 95% CI, 7.73–11.43) than the non-cancerous organ tissues analyzed via qPCR.

### 3.4 Mesothelin expression in the clinical cancer tissues

**Figure 4:**
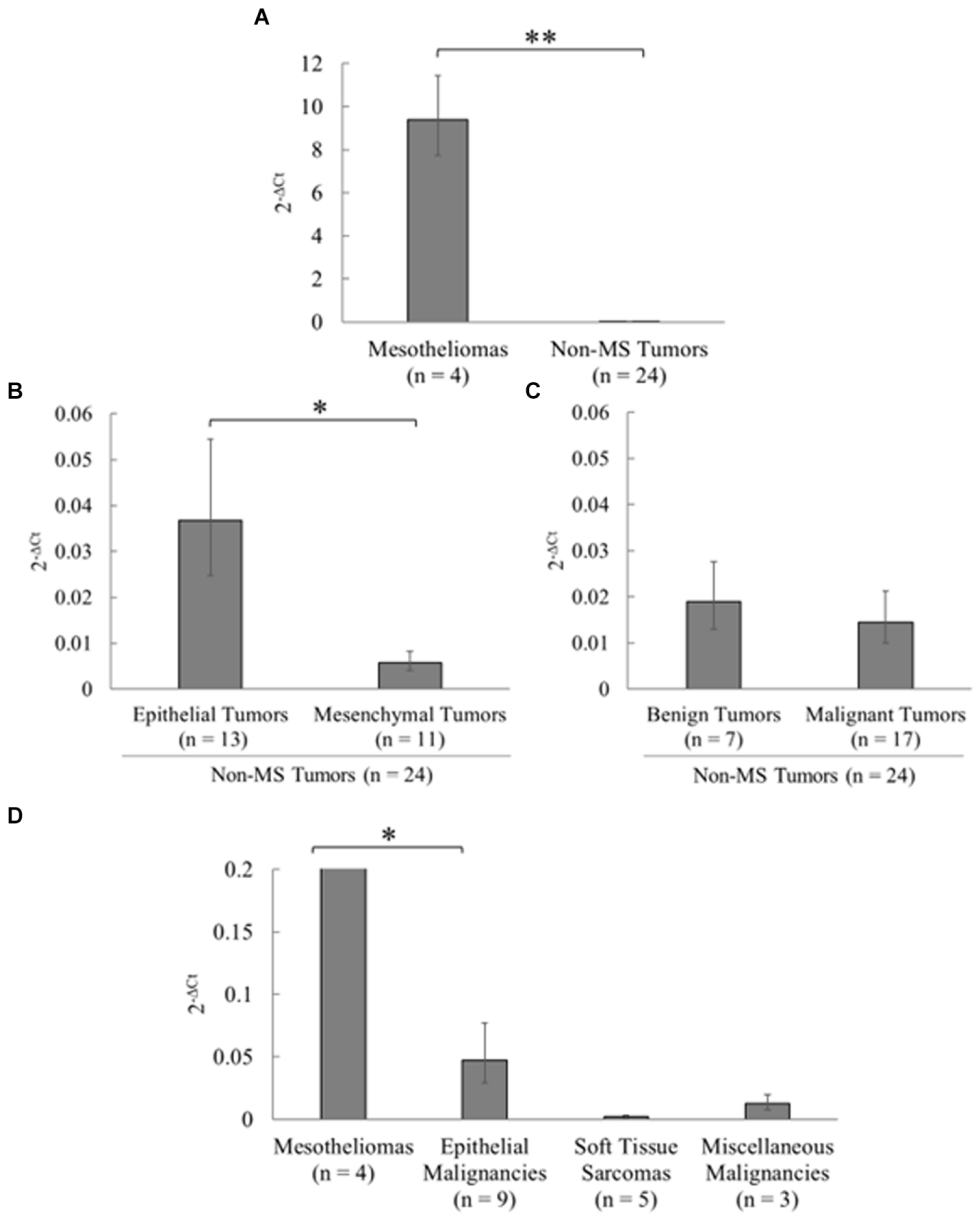
Analysis of mesothelin expression in various tumors in dogs. Mesothelin expression in various canine tumors was examined using quantitative polymerase chain reaction (qPCR). **(A)** Significantly higher mesothelin levels were detected in the mesotheliomas than in non-mesothelioma (non-MS) tumors (*p* < 0.001). **(B)** Among the non-MS tumors, epithelial tumors expressed significantly higher mesothelin levels than mesenchymal tumors (*p* = 0.045). **(C)** Malignant tumors exhibited mesothelin levels similar to those in the benign tumors (*p* = 0.656). **(D)** When comparing malignant tumors, epithelial malignancies showed relatively higher expression levels than soft tissue sarcomas and other malignant tumors. Mesotheliomas exhibited greater overexpression compared to the non-MS malignancies.

Relative expression levels were compared between mesothelioma and non-mesothelioma (non-MS) tumor tissues using qPCR to analyze mesothelin expression in various tumor types (Figure 4; Supplementary Table S3). Consequently, significantly higher mesothelin expression was detected in the mesotheliomas (mean, 9.40; 95% CI, 7.73–11.43) than in the non-MS tumors (mean, 0.016; 95% CI, 0.012–0.021) (*p* < 0.001). Mesothelin expression between epithelial and mesenchymal tumors, and benign and malignant tumors, were compared to investigate the potential relationship between mesothelin expression and the cell of origin, and its expression and malignant nature of tumors, respectively. Epithelial tumors (mean, 0.037; 95% CI, 0.025–0.054) expressed significantly higher mesothelin levels than mesenchymal tumors (mean, 0.006; 95% CI, 0.004–0.008) (*p* = 0.045). Among the epithelial tumors examined, both cases (2/2) of apocrine gland adenocarcinoma of the anal sac showed higher mesothelin gene expression (0.35 and 1.37). Malignant tumors (mean, 0.015; 95% CI, 0.010–0.021) exhibited similar mesothelin levels as the benign tumors (mean, 0.019; 95% CI, 0.013–0.028) (*p* = 0.656). When comparing malignant tumors, epithelial malignancies showed relatively higher expression levels than soft tissue sarcomas and other malignant tumors. However, statistical analysis did not reveal significant differences among the three non-MS malignancies, likely due in part to the low number of cases in each group. Mesotheliomas exhibited greater overexpression compared to the non-MS malignancies.

### 3.5 Mesothelin expression in the clinical effusion samples

**Figure 5:**
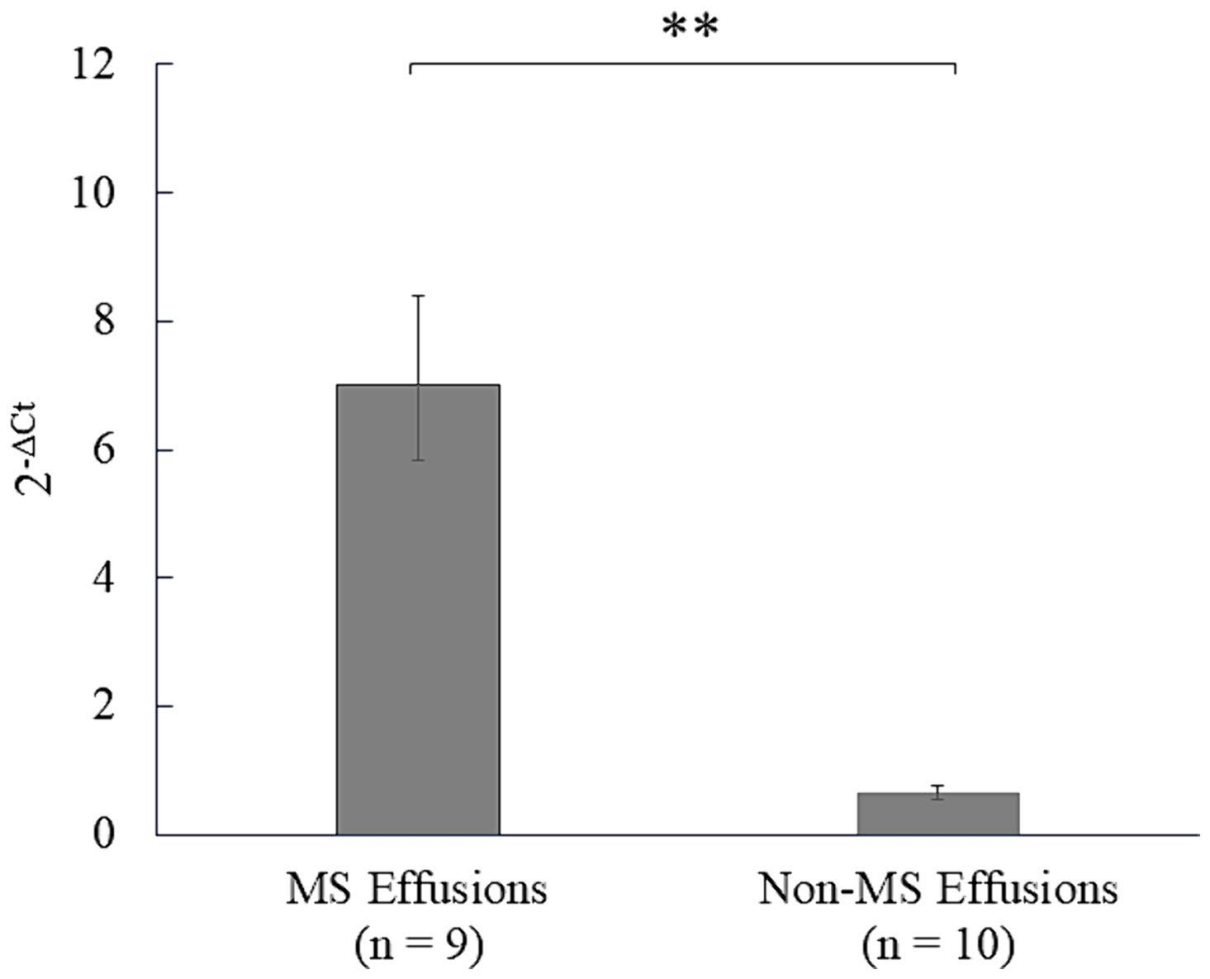
Analysis of mesothelin expression in the effusions in dogs. Mesothelin expression in the clinical effusion samples was analyzed using quantitative polymerase chain reaction (qPCR). Mesothelin was overexpressed in the mesothelioma (MS) effusions compared to the non-mesothelioma (non-MS) effusions (*p* < 0.001), underscoring the diagnostic utility of mesothelin expression analysis in canine mesothelioma effusions.

Subsequently, mesothelin expression was analyzed using clinical effusion samples, considering effusion as the major clinical sign by which patients are presented at a veterinary clinic (Figure 5; Supplementary Table S3). Significantly higher mesothelin expression was detected in the effusions from mesotheliomas (mean, 6.99; 95% CI, 5.82–8.40) than those from the non-mesotheliomas (mean, 0.65; 95% CI, 0.55–0.77) (*p* < 0.001). Lymphoma effusions exhibited the highest mesothelin expression among the non-mesothelioma effusions (2.63).

## 4 Discussion

Mesothelin is a GPI-anchored cell surface glycoprotein that was initially identified and recognized by the monoclonal antibody Mab K1 (8, 10, 24). In humans, mesothelin is normally expressed at low levels on the mesothelial cell surface and is overexpressed in numerous cancers, including mesothelioma (8, 12–15, 24). Herein, mesothelin gene expression in canine mesothelioma was first assessed via conventional RT-PCR, followed by sequence analysis of cultured canine pericardial mesothelioma cells. These results indicated that the mesothelin gene is expressed in the canine mesothelioma, similar to that in humans. A database search showed nine splicing variants of the canine mesothelin and seven isoforms with variants 7–9 coding for the same isoform 7. Canine mesothelin splicing variants are likely a product of 3′ or 5′ splice sites alternatively selected. This type of alternative splicing is believed to lead to minor changes in the coding product, creating diversity (25). A single peak was observed in the chromatogram of a canine mesothelioma samples despite the presence of splicing variants, six of which were targeted by RT-PCR. These discordant results might present clues for determining biologically important variants in cancer biology in dogs. Further studies are warranted to validate these hypotheses.

The predicted amino acid sequence of the canine mesothelin was compared with that of the human mesothelin after confirming mesothelin expression in the canine mesothelioma via RT-PCR. Considerable similarities were observed where mature mesothelins are encoded. Human mesothelin is initially expressed as a preproprotein and becomes a mature membrane-bound mesothelin through furin protease cleavage, leaving the C-terminal region bound to the cell membrane via a GPI-anchor. Comparing the amino acid sequences of human and canine mesothelin implied that canine mesothelin may have a putative signal peptide, furin cleavage site, disulfide bond, glycosylation residues, and a GPI-anchorage attachment site (26, 27). Moreover, amino acids crucial for heterotopic binding to CA125 in humans (tyrosine^318^, tryptophan^321^, and glutamic acid^324^) are conserved in canine mesothelin (28). The biological function of mesothelin in normal tissues remains to be understood because a previous study did not detect any anatomical or histological abnormalities in mesothelin gene-knockout mice (29). However, potential roles in cell adhesion have been noted (8) and various potential roles in tumorigenesis and progression in mesothelin-expressing cancers have been revealed (18, 30–34). The binding of CA125 to mesothelin is a key step in downstream cascade activation (30, 31, 35–40). Canine mesothelin may play a role similar to human mesothelin in cancer biology because these structurally and functionally important regions of human mesothelin are conserved in canine mesothelin.

Additionally, comparative sequence analysis revealed canine mesothelin’s unique features. Canine mesothelin lacks the N-terminus corresponding to human MPF. This was an unexpected finding, considering that sequence analysis of murine mesothelin showed a predicted amino-terminal fragment potentially released from a precursor protein by furin cleavage, corresponding to human MPF (27). Human MPF is a soluble protein initially purified from the culture supernatants of human pancreatic cancer cells (41, 42). The study group revealed that human MPF was substantially expressed in the lungs and exhibited megakaryocyte-potentiating activity in the presence of murine interleukin-3 in an *in vitro* assay with mouse bone marrow cells. However, its biological function remains unclear as the knockout mice exhibited normal platelet counts (29, 43). Interestingly, a previous comparative study revealed that cows, pigs, horses, pandas, hedgehogs, and dogs, all of which belonging to Laurasiatherian mammals, lacked a putative MPF portion, hypothetically because of the non-homologous end-joining of the introns during Laurasiatherian mammal evolution (44). Contrastingly, euarchontogliran members, such as humans and mice, retained the ancestral *MSLN* gene, including the MPF portion. The biological significance of this segmental exon deletion is uncertain; however, the relatively high MPF expression in the lungs of humans compared to other organs is intriguing because the lungs have been historically considered a potentially important site for thrombopoiesis, in addition to the bone marrow. Megakaryocytes released from the bone marrow or spleen have recently been shown to reside in the lungs and have the capacity to repopulate the bone marrow to reconstitute platelets in the circulation during thrombocytopenia (45, 46). MPF expression in the lungs possibly exhibits beneficial effects on the prompt recovery from thrombocytopenia via accelerated megakaryocytopoiesis/thrombopoiesis in the lungs of Euarchontogliran species that retain a functional MPF portion of the *MSLN* gene. This hypothesis may explain why knockout mice did not exhibit apparent abnormalities because the bone marrow could function as a primary site for megakaryocytopoiesis/thrombopoiesis in the presence of other major stimulatory cytokines. Further investigations are necessary to confirm this theory.

One of the characteristics of mesothelin is its limited expression in healthy organs and overexpression in mesotheliomas. We subsequently characterized the distribution of mesothelin expression in dogs using qPCR given the molecular similarities between canine and human mesothelin. qPCR was designed to cover all variants considering the presence of canine mesothelin variants and the lack of knowledge regarding biologically active or predominant isoforms in dogs. Therefore, it can be used to evaluate changes in the overall mesothelin gene expression, regardless of which variant is biologically crucial. Mesothelin expression was low in the mesothelial and lung tissues, whereas only negligible expression was observed in the other major organ tissues. Mesothelin was overexpressed in mesotheliomas. This unique mesothelin expression pattern is consistent with that in humans (8, 24, 42). Mesothelin has attracted attention in human oncology as a promising candidate for a novel cancer immunotherapy target owing to its limited expression in healthy organs and its overexpression in mesotheliomas (10, 47). Conventional cancer treatments, such as chemotherapy, cannot avoid notorious effects on healthy cells, which often limit clinical outcomes. To address this therapeutic dilemma, cancer immunotherapy targets tumor-associated antigens such as mesothelin to have maximal effects on cancer cells with minimal effects on the healthy cells. Various mesothelin-targeted therapies in ongoing clinical trials, including monoclonal antibodies, immunotoxins, antibody-drug conjugates, vaccines, and chimeric antigen receptor T (CAR-T) cells have been studied (10, 37, 48–51). Canine mesotheliomas could be an ideal model for assessing mesothelin-targeted therapies for human mesotheliomas since canine mesothelin shares a similar expression pattern. Additionally, it would be interesting to examine the potential efficacy of mesothelin-targeted immunotherapies for canine mesotheliomas.

We evaluated mesothelin’s expression in non-mesothelioma tumor tissues to further assess its potential value as a diagnostic biomarker. The results demonstrated substantial mesothelin overexpression in the mesotheliomas compared to that in the non-mesothelioma tumors, supporting the mesothelin’s potential diagnostic usefulness in canine mesotheliomas. Further studies are needed to confirm the findings given the limited number of mesothelioma cases involved in the study. Low mesothelin expression was found in a few epithelial tumors, including the apocrine gland adenocarcinoma of the anal sac and mammary gland tumors, whereas none of the mesenchymal tumors expressed substantial mesothelin levels. This was not unexpected given that cell adhesion is considered a major mesothelin function (8). Additionally, this is akin to human oncology, in which several tumors of epithelial origin exhibit aberrant mesothelin expression (8, 12–15). No considerable difference in mesothelin expression was observed between benign and malignant non-mesothelioma tumors, suggesting that mesothelin overexpression is not necessary for the malignant nature of non-mesothelioma tumors.

Finally, given that dogs with mesotheliomas almost always present with effusion, mesothelin expression was analyzed using clinical effusion samples. Effusion collection is an easy, minimally invasive, and well-tolerated procedure that is often performed at private clinics for diagnostic and palliative purposes. Additionally, mesothelioma cells can be exfoliated from the original tumors in an active state with a nutritious supply from the effusion (3, 52, 53). Thus, assessing the diagnostic usefulness of mesothelin in effusion samples was plausible, with the hope that it could be easily introduced in clinical settings. Mesothelin overexpression was observed in mesothelioma effusions, suggesting the effusion samples’ diagnostic usefulness for analyzing mesothelin expression in canine mesothelioma. Differentiating between neoplastic and reactive mesothelial cells is one of the effusion-based diagnostic dilemmas of mesothelioma in clinical settings because reactive mesothelial cells can exhibit an atypical morphological appearance upon simulation, overlapping with neoplastic cells (4, 54–57). To complicate the problem, neoplastic mesothelial cells may not display apparent morphological malignancy features (56, 57). Therefore, gene expression analysis is possibly an ideal additional test to complement morphological assessments.

In conclusion, canine and human mesothelin share molecular and biological similarities. This preliminary study suggests that mesothelin could serve as a vital diagnostic biomarker of canine mesotheliomas for tissue- and effusion-based samples. Further studies are warranted to confirm the findings. Notably, this unique expression pattern makes mesothelin an attractive therapeutic target for immunotherapies, and further investigation of its efficacy as a novel treatment option for canine mesothelioma is necessary.

## Data availability statement

The original contributions presented in the study are included in the article/Supplementary material, further inquiries can be directed to the corresponding author.

## Ethics statement

The animal studies were approved by the Tokyo University of Agriculture and Technology Animal Experiment Committee (approval No. 31–2) and Clinical Research Ethics Committee of the Tokyo University of Agriculture and Technology (approval No. 0016017, 0020005, 00500011). The studies were conducted in accordance with the local legislation and institutional requirements. Written informed consent was obtained from the owners for the participation of their animals in this study.

## Author contributions

RN: Conceptualization, Data curation, Formal analysis, Investigation, Methodology, Resources, Software, Validation, Visualization, Writing – original draft, Writing – review & editing. AK: Data curation, Formal analysis, Resources, Writing – review & editing. KS: Data curation, Formal analysis, Resources, Writing – review & editing. KM: Data curation, Formal analysis, Resources, Writing – review & editing. AY: Data curation, Formal analysis, Methodology, Resources, Writing – review & editing. TO: Data curation, Formal analysis, Methodology, Resources, Writing – review & editing. DA: Data curation, Formal analysis, Resources, Writing – review & editing. TF: Conceptualization, Data curation, Formal analysis, Investigation, Methodology, Resources, Software, Supervision, Validation, Visualization, Writing – original draft, Writing – review & editing. TU: Conceptualization, Data curation, Funding acquisition, Investigation, Methodology, Project administration, Resources, Supervision, Validation, Visualization, Writing – original draft, Writing – review & editing.
