# Supplementary figures and images for: Characterization of mesothelin gene expression in dogs and overexpression in canine mesotheliomas
Source: Front Vet Sci. 2024 Sep 9;11:1436621. doi: 10.3389/fvets.2024.1436621 (PMC11417096; doi:10.3389/fvets.2024.1436621)

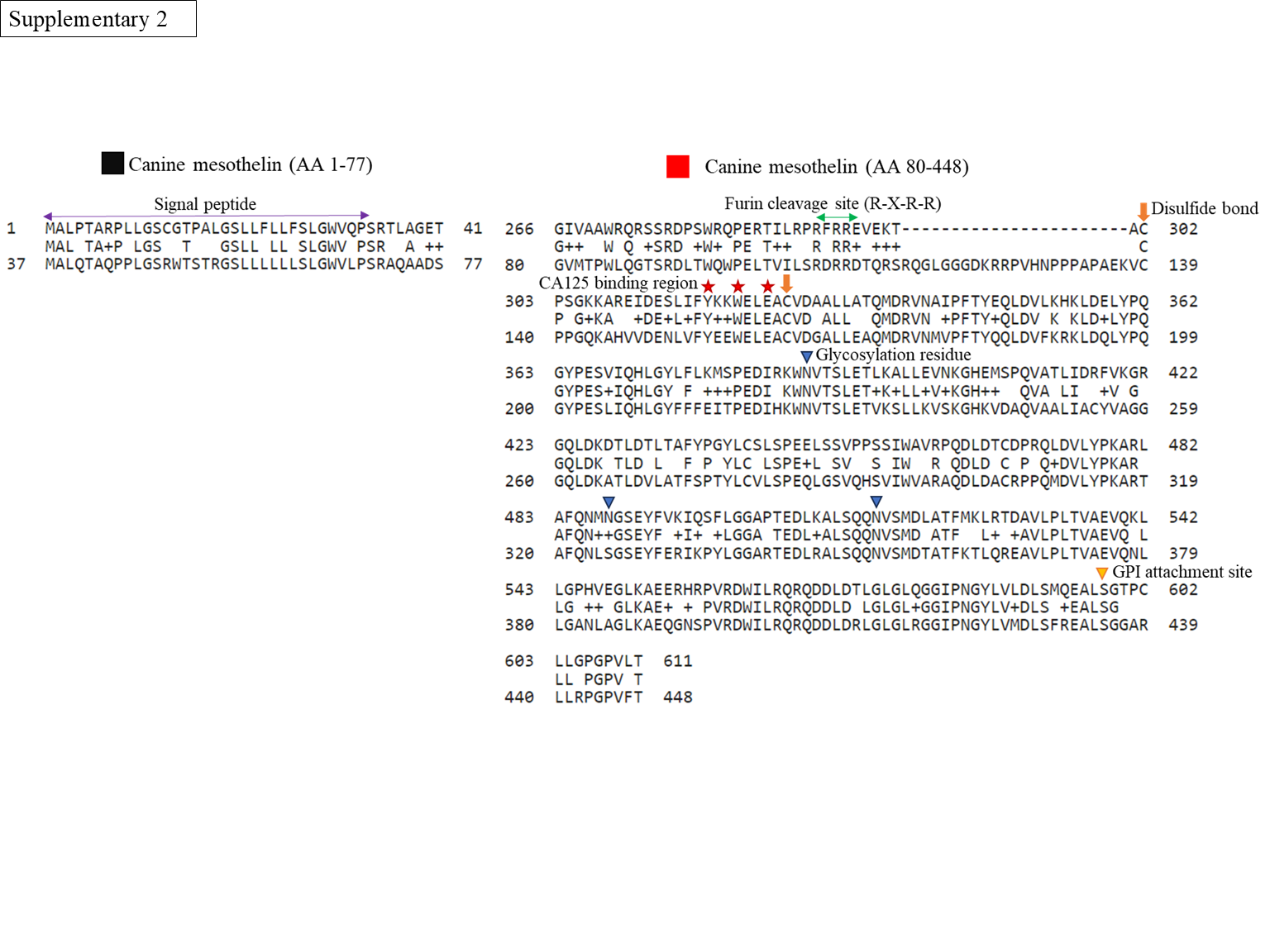

Supplement: Supplementary file 3 [file Image_1.tif]
